# Acupuncture and related acupoint therapies for smoking cessation: An umbrella review and updated meta-analysis

**DOI:** 10.18332/tid/186147

**Published:** 2024-04-18

**Authors:** Ying-Ying Zhang, You-Zhu Su, Zi-Yu Tian, Shi-Bing Liang, Yi-Jie Liu, Yu-Fei Li, Hai-Fa Qiao, Nicola Robinson, Jian-Ping Liu

**Affiliations:** 1 College of Acupuncture-Moxibustion and Tuina, Shaanxi University of Chinese Medicine, Xianyang, China; 2 Key Laboratory of Acupuncture and Medicine in Shaanxi Province, Xianyang, China; 3 Centre for Evidence-Based Chinese Medicine, Beijing University of Chinese Medicine, Beijing, China; 4 Institute of Acupuncture and Moxibustion, China Academy of Chinese Medical Sciences, Beijing, China; 5 Clinical Study Center, Affiliated Hospital of Shandong University of Traditional Chinese Medicine, Jinan, China; 6 Institute of Health and Social Care, London South Bank University, London, United Kingdom

**Keywords:** acupuncture, acupressure, smoking cessation, umbrella review, meta-analysis

## Abstract

**INTRODUCTION:**

Acupuncture and related acupoint therapies have been widely used for smoking cessation. Some relevant systematic reviews (SRs) have been published. There is a need to summarize and update the evidence to inform practice and decision-making.

**METHODS:**

Eight databases were searched from their inception to December 2023. SRs, any randomized controlled trials (RCTs) comparing acupuncture therapies with sham acupuncture, pharmacotherapy, behavioral therapy, or no treatment, were included. The primary outcome was the abstinence rate. AMSTAR-2 was employed to assess the quality of SRs. An updated meta-analysis was conducted based on SRs and RCTs. Data were synthesized using risk ratios (RR) with 95% confidence intervals (CIs). The GRADE approach was employed to assess the certainty of the updated evidence.

**RESULTS:**

Thirteen SRs and 20 RCTs outside of the SRs were identified. The SRs were of low or very low quality by AMSTAR-2. Sixteen (80%) RCTs were at high risk of performance bias. Eight acupuncture and related acupoint therapies were involved. The short-term (≤6 months) abstinence rate outcome was summarized as follows. Most SRs suggested that filiform needle acupuncture or acupressure had a better effect than sham acupuncture, but the findings were inconsistent. The updated meta-analysis also suggested that filiform needle acupuncture was more effective than sham acupuncture (RR=1.44; 95% CI: 1.02–2.02; I^2^ = 66%; low certainty; 9 RCTs, n=1358). Filiform needle acupuncture combined with acupressure was comparable to nicotine patches (RR=0.99; 95% CI: 0.74–1.32; low certainty; 6 RCTs, n= 524). Acupressure was superior to counseling (RR=1.46; 95% CI: 1.14–1.87; I^2^=5%; low certainty; 8 RCTs, n=595). No serious adverse events were reported in these SRs or RCTs.

**CONCLUSIONS:**

Low certainty evidence suggests that filiform needle acupuncture and auricular acupressure appear to be safe and effective in achieving short-term smoking cessation. However, long-term follow-up data are needed.

## INTRODUCTION

Cigarette smoking is ranked as the second most important risk factor for deaths globally^1^. Reports suggest that at least 8 million people die from smoking every year worldwide^1,2^. Smoking is associated with cardiovascular and respiratory diseases, cancer, and many other debilitating diseases. China is a major tobacco producing and consuming country, and it is estimated that the smoking-attributed disease burden in China will increase further unless active smoking cessation interventions are implemented^3,4^. Currently, the most effective smoking cessation therapy recommended in guidelines is pharmacological therapy combined with behavioral intervention^5,6^. However, pharmacological treatments, including nicotine replacement therapy (NRT), varenicline, and bupropion, have limited use in quitting due to their high cost, side effects, and low popularity^7,8^.

Acupuncture has been used for smoking cessation for more than 40 years in different countries^9,10^. Acupuncture has been recommended as a complementary and alternative approach for quitting smoking in guidelines; however, it is recognized that further high-quality evidence is needed^5^. Clinical and experimental studies suggest that acupuncture promotes the release of endogenous opioids to relieve withdrawal symptoms^11^ or suppress the craving for cigarettes after quitting smoking^12^. Historically, traditional acupuncture has generally used filiform needles that penetrate the skin and are manipulated to stimulate acupoints. However, with the development of acupuncture techniques, other acupuncture and related acupoint stimulation therapies have also been used for smoking cessation due to convenience and to enhance compliance, such as acupressure^13^, transcutaneous electrical acupoint stimulation (TEAS)^14^, laser acupuncture^15^, and acupoint catgut embedding (ACE)^16^.

Many systematic reviews (SRs)^10,17-19^ on acupuncture therapies have been published. Some SRs^10,17^ have suggested that there was insufficient evidence to confirm the efficacy of acupuncture therapies for smoking cessation. However, the latest SRs^18,19^ suggest that filiform needle acupuncture and acupressure may have significant benefits in achieving smoking cessation. In addition to the contradictory findings between these SRs, acupuncture interventions evaluated in these SRs were different and incomplete. It has been acknowledged that umbrella reviews are a valuable tool for clinical decision-making since they avoid uncertainty induced by contradictory conclusions from different SRs and also provide a broader picture of many treatments^20^. Therefore, in this study, an umbrella review was conducted to provide an evidence profile of acupuncture and related acupoint therapies for smoking cessation.

## METHODS

This umbrella review was performed according to the Cochrane Handbook for conducting overviews^21^ and the methodological process for an umbrella review from Joanna Briggs Institute^20^. The umbrella review was reported following PRISMA 2020 checklist^22^. The updated meta-analysis was based on all available RCTs that have been evaluated in the included SRs and unevaluated RCTs. The protocol of this umbrella review was registered on INPLASY prospectively (INPLASY202410106)^23^.

### Eligibility criteria

The study types included SRs and randomized controlled trials (RCTs). The eligible SRs were based on RCTs or quasi-RCTs. The study population was adult cigarette smokers (aged ≥18 years) regardless of gender, ethnicity, and health status. The eligible tobacco product used by smokers was conventional cigarettes. The eligible interventions were acupuncture or acupuncture-related therapies, including traditional acupuncture with filiform needles penetrating the skin to stimulate the acupoints with or without manipulations, and other acupoint stimulation therapies that were not stimulated by the needle but use other acupuncture techniques, such as acupressure, Chinese herbal medicine external use at acupoints, transcutaneous electrical acupoint stimulation (TEAS), laser acupuncture, intradermal needle, fire acupuncture, and acupoint catgut embedding (ACE). The eligible comparisons were no intervention, placebo, pharmacotherapy (NRT, bupropion, or varenicline), behavioral counseling, or sham acupuncture. The primary outcome was a continuous abstinence rate defined as smoking cessation between a quit day and a follow-up period. Smoking cessation could be self-reported or biochemically validated. The secondary outcomes were adverse events.

### Search strategy

We systematically searched SRs and RCTs from PubMed, the Cochrane Library, EMBASE, Web of Science, China National Knowledge Infrastructure (CNKI), Chinese Scientific Journal Database (VIP), Sino-Med and Wanfang databases from their inception to 20 December 2023. The search strategy for each database is presented in the Supplementary file (together with a graphical abstract shown as Supplementary file Figure 1).

### Study selection and data extraction

The literature was managed by Note Express (3.2.0.7535). After removing duplicates, two reviewers (YZS, SBL) independently screened studies by title and abstract. Uncertainty was determined for eligibility by checking full texts. Reasons for excluding SRs were recorded at the full-text screening stage, and any discrepancies were discussed by two review authors and arbitrated if required by a third party (JPL). In the data extraction process, data were extracted by two review authors (YZS, SBL) independently using a pre-defined electronic data extraction form, which included basic information, objectives, details of participants, interventions, comparisons, and outcomes.

### Quality appraisal

The methodological quality of SRs and RCTs was evaluated independently by two review authors (YJL, YFL). A measurement tool to assess SRs (AMSTAR-2)^24^ was employed to evaluate the quality of the included SRs. AMSTAR-2 has 16 items with seven key items, and each of the 16 items can be assessed as ‘yes’, ‘no’, or ‘partially yes’, according to whether the review was appropriately conducted in line with the items. The overall confidence for each systematic review was evaluated as high, moderate, low, or critically low. Cochrane Risk of Bias tool (ROB)^25^ was employed to assess the methodological quality of RCTs from seven domains (random sequence generation, allocation concealment, blinding of participants and personnel, blinding of outcome assessment, incomplete outcome data, selective reporting, and other biases). RCTs could be assessed as low, high, or unclear risk of bias in each domain. Discrepancies were resolved by discussion or judged by a third party (JPL).

### Data synthesis and analysis

The types of acupuncture and related acupoint therapies that were classified included SRs and RCTs. The findings from SRs were synthesized narratively. The updated meta-analysis was based on all available RCTs that have been evaluated in the included SRs and unevaluated RCTs. Data are presented as risk ratios (RR) with 95% confidence intervals (CIs). Meta-analysis was conducted using Cochrane Review Manager 5.4 software, and I^2^ statistics were utilized to test the statistical heterogeneity^25^. According to the Cochrane Handbook for Systematic Reviews of Interventions^25^, the fixed effects model was applied when I^2^ ≤30%, which represented low heterogeneity among the included trials in each meta-analysis, otherwise the random effects model was used when the heterogeneity was moderate (30%< I^2^ ≤ 75%) or substantial (I^2^ >75%). We predefined the subgroup analysis: 1) by the duration of follow-up, short-term (<6 months) versus long-term (≥6 months); 2) by means of abstinence rate measures, self-reported versus biologically validated; and 3) by the comparisons, active control versus inactive control, active controls refer to conventional therapy alone or conventional therapy plus sham acupuncture, inactive controls refer to sham acupuncture or no treatment. Funnel plots were generated to detect possible publication bias if ≥10 RCTs were included in a meta-analysis.

### Certainty of evidence

GRADE^26^ (Grading of Recommendations Assessment, Development and Evaluation) approach was employed to evaluate the certainty of evidence from the updated meta-analysis in five domains (risk of bias, directness, precision, consistency, and the possibility of publication bias). Two reviewers (YJL, YFL) independently assessed the quality of evidence from the updated meta-analysis.

## RESULTS

### Screening

**Figure 1:**
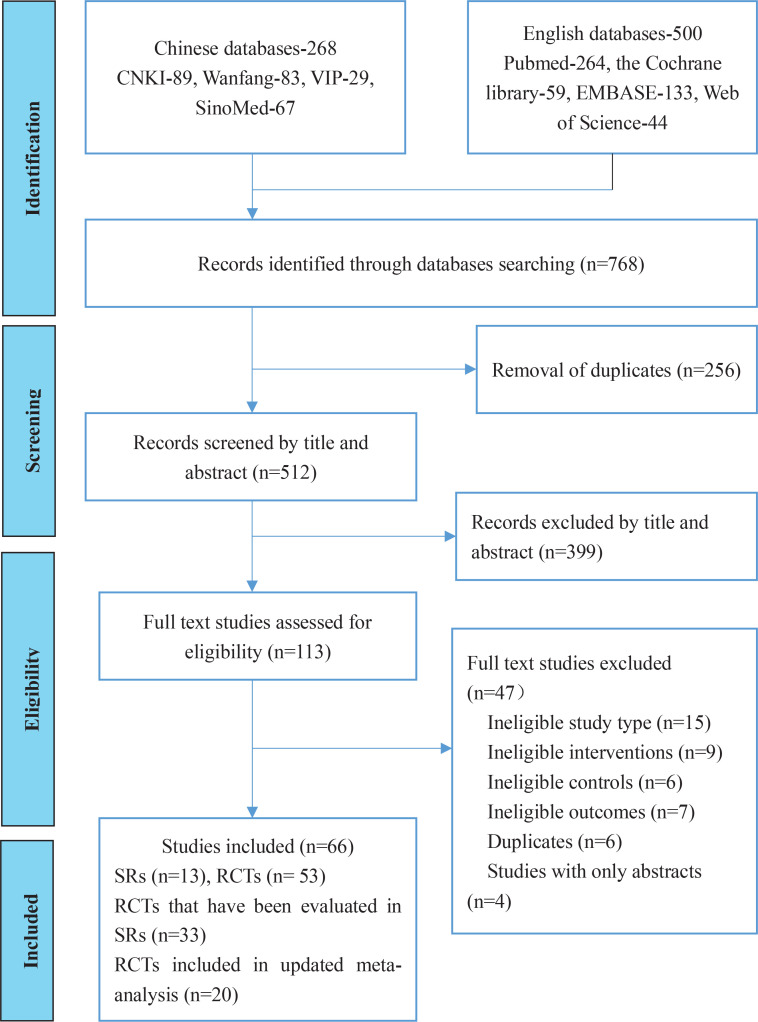
Flow chart of study selection

Initially, 768 records were retrieved and 256 duplicates were removed. In all, 399 records were excluded by scanning the title and abstract. This left 113 records, of which 47 studies were excluded through full-text screening due to ineligible study type, ineligible interventions, ineligible controls or outcomes, duplicates, or lack of outcomes. Finally, 13 SRs and 53 RCTs were included after full-text screening. Of these 53 RCTs, 33 RCTs had been evaluated in the included 13 SRs, and 20 RCTs were outside of these SRs. The screening process is shown in Figure 1.

### Systematic reviews

A total of 13 SRs^10,17-19,27-35^ on acupuncture and related acupoint therapies were included; all of these SRs were based on RCTs or quasi-RCTs. The characteristics of the included SRs are shown in Table 1. We classified these SRs into different categories according to the acupuncture techniques used. There were 5 SRs^10,17,27-29^ which compared the effects of various acupuncture techniques with sham acupuncture, no treatment, NRT, or behavioral counseling for smoking cessation regardless of the type of acupuncture performed, such as traditional filiform needle acupuncture, acupressure, TEAS, intradermal needle, or laser acupuncture. The acupoints chosen could be on the body, ear, head, or wrist. Three SRs^30-32^ focused on auricular acupuncture, including auricular acupressure, auricular needle acupuncture, or auricular laser acupuncture. Another three reviews^33-35^, also examined the effect of body filiform needle acupuncture combined with auricular acupuncture for smoking cessation. One review^18^ only evaluated the transdermal body acupuncture by filiform needles for smoking cessation. The remaining single review^19^ assessed the effect of non-traditional filiform needle acupuncture therapies for smoking cessation, such as acupressure, laser acupuncture, TEAS, or acupoint catgut embedding (ACE). In terms of the synthesis methods, only one systematic review^33^ was narratively described. The remaining 12 SRs were all quantitatively synthesized. The study protocol was prospectively registered in three SRs^19,29,31^. Only one systematic review^10^ provided the list of excluded studies. GRADE approaches were employed to evaluate the certainty of evidence in 2 reviews^10,19^, and trial sequential analyses were conducted to detect the robustness of the results in one review^18^. The outcomes reported in these SRs were mainly abstinence rate, nicotine dependence, or withdrawal symptoms.

**Table 1:** Thirteen systematic reviews of acupuncture and related acupoint therapies for smoking cessation

*Authors Year*	*Number of included RCTs*	*Interventions (I)*	*Comparisons (C)*	*Methods of synthesis*	*Authors’ conclusions*	*Study protocol*	*Certainty of evidence assessed by GRADE*	*AMSTAR-2 rating (n/16)*
**Acupuncture and related acupoint therapies**								
White et al.^17^ 1999	14	Acupuncture (no restrictions on acupuncture techniques.	Sham acupuncture, no treatment, counseling, or pharmacotherapy.	Quantitative synthesis-meta-analysis	Acupuncture was not superior to sham acupuncture for smoking cessation; acupuncture technique was not associated with a positive effect.	No	No	Critically low (3/16)
Cheng et al.^27^ 2012	20	Filiform needle acupuncture, acupressure, electro-acupuncture, intradermal needle, laser acupuncture.	Sham acupuncture, no treatment, counseling, or pharmacotherapy.	Quantitative synthesis-meta-analysis	Acupuncture combined with counseling or other interventions, may help smokers quit smoking and prevent relapse.	No	No	Critically low (7.5/16)
White et al.^10^ 2014	38	Filiform needle acupuncture, acupressure, TEAS, laser acupuncture, intradermal needle.	Sham acupuncture, no treatment, counseling, or pharmacotherapy.	Quantitative synthesis-meta-analysis	Although pooled estimates suggest possible short-term effects, there is no consistent, bias-free evidence that acupuncture have a long-term benefit on cessation.	No	Yes	Moderate (12/16)
Liu et al.^28^ 2015	24	Filiform needle acupuncture, laser acupuncture, acupressure, wrist-ankle acupuncture, electro-acupuncture.	Sham acupuncture, placebo, NRT, or counseling.	Quantitative synthesis-meta-analysis	Acupuncture has positive advantages on shortterm smoking cessation; however, its long-term effect needs to be verified.	No	No	Critically low (5.5/16)
Dai et al.^29^ 2021	23	Filiform needle acupuncture, acupressure, or acupuncture combined with acupressure.	Sham acupuncture, NRT, or auricular acupressure alone.	Quantitative synthesis-network meta-analysis	Auricular acupressure was superior to sham acupressure for smoking cessation, but there was no significant difference for long-term cessation.	Yes	No	Critically low (10/16)
**Auricular acupressure**								
White et al.^30^ 2006	13	A semi-permanent acupuncture needle or device (needle, bead or suture) placed in the ear.	Pharmacotherapy, sham acupuncture on ‘incorrect’ points.	Quantitative synthesis-meta-analysis	Auricular acupuncture appears to be effective for smoking cessation, but the effect may not depend on point location.	No	No	Critically low (5/16)
Tahiri et al.^31^ 2012	14 Of these, 6 on acupuncture	Acupuncture, electrotherapy, laser therapy on the ear.	Sham acupuncture	Quantitative synthesis-meta-analysis	Acupuncture may help smokers quit smoking. However, there are no recent trials investigating this intervention.	Yes	No	Critically low (6/16)
Di et al.^32^ 2014	25	Auricular needle acupuncture or acupressure or other types of auricular-therapy.	Sham acupuncture, placebo, no treatment, wait-list, or other ‘inactive’ control. Medical or behavioral therapies. Body acupuncture.	Quantitative synthesis-meta-analysis	Acupressure was superior to sham controls for short-term smoking cessation. However, this effect was not observed at long-term follow-up.	No	No	Critically low (9/16)
**Filiform needle acupuncture + auricular acupressure**								
Kim et al.^33^ 2012	3 On acupuncture and acupressure	Pharmacotherapy, counseling, or TCM (e.g. acupuncture, auricular acupressure).	Not reported	Qualitative analysis-narrative review	More RCTs of TCM approaches and physician advice are needed with long-term follow-up assessments.	No	No	Critically low (5.5/16)
Liu et al.^34^ 2019	9	Acupuncture, auricular acupressure, or Chinese herbal medicine external use at acupoints.	Chinese herbal medicine, or NRT patches.	Quantitative synthesis-meta-analysis	Filiform needle acupuncture combined with acupressure was comparable to NRT in smoking cessation.	No	No	Critically low (5.5/16)
Kuang et al.^35^ 2022	16	Acupuncture or auricular acupressure used alone or combined with other treatments.	Treatments other than acupuncture and acupressure.	Quantitative synthesis-meta-analysis	Acupuncture or acupressure may improve abstinence rate, reduce the degree of nicotine dependence, and relieve withdrawal symptoms.	No	No	Critically low (9/16)
**Filiform needle acupuncture**								
Wang et al.^18^ 2019	24	Filiform needle acupuncture (either alone or in conjunction with other interventions).	No intervention, waiting list, placebo, or other interventions.	Quantitative synthesis-meta-analysis	Acupuncture combined with counseling or educational smoking cessation program, was more effective than acupuncture alone for long-term smoking cessation.	No	No	Critically low (9/16)
**Non-traditional filiform needle acupuncture therapies**								
Zhang et al.^19^ 2021	25	Acupressure, TEAS, laser acupuncture, intradermal needle, or acupoint catgut embedding.	Sham acupuncture, pharmacotherapy, counseling, or no treatment.	Quantitative synthesis-meta-analysis	Low-certainty evidence suggests that non-traditional acupuncture therapies were effective in achieving short-term smoking cessation.	Yes	Yes	Low (14/16)

AMSTAR: a measurement tool to assess systematic reviews. CHM: Chinese herbal medicine. GRADE: grading of recommendations assessment, development and evaluation. RCT: randomized controlled trial. NRT: nicotine replacement therapy. S: study type. TEAS: transcutaneous electrical acupoints stimulation. TCM: traditional Chinese medicine.

### Randomized controlled trials

Fifty-three RCTs were included after full-text screening, of which 33 RCTs were evaluated in the 13 SRs that were included. Therefore, 20 RCTs^34,36-54^ involving 3532 participants were included. The characteristics are shown in Table 2. The sample size ranged from 41 to 900, and the study population was healthy adult smokers, except in one review^42^, including smokers with angina pectoris. Six RCTs^34,36-40^ compared the effect of Chinese herbal medicine - external use at acupoints with behavioral counseling, nicotine patches, or placebo for smoking cessation. Chinese herbal medicine patches were usually applied on Tim-Mee acupoint, which was a newly discovered acupoint located on the wrist only for smoking cessation. Two RCTs^41,42^ focused on auricular acupressure, and two RCTs^42,43^, compared the effect of auricular acupressure with nicotine patches or no treatment. Body filiform needle acupuncture alone or combined with auricular acupressure was used in 6 RCTs^43-48^. There was one RCT^49^ on fire needle acupuncture, 3 RCTs^50-52^ on TEAS, and 2 RCTs^53,54^ on laser acupuncture. Tim-Mee, Lieque (LU7), and Hegu (LI4) were the most commonly used body acupoints for smoking cessation. Lung (CO_14_), Shenmen (TF_4_), and Mouth (CO_1_) were the most commonly used auricular acupoints. In terms of outcomes, abstinence rate, nicotine dependence, and withdrawal symptoms were usually reported. However, the abstinence rate was not reported in 6 RCTs^38,39,50,52-54^; therefore, we were unable to pool these data.

**Table 2:** RCTs of acupuncture and related acupoint therapies for smoking cessation (N=20)

*Authors Year*	*Study population & Sample size (I/C)*	*Intervention (I)*	*Comparison (C)*	*Treatment duration*	*Outcomes reported*	*Trial registration*
**Chinese herbal medicine - external use at acupoints**						
Gu et al.^36^ 2007	Adult smokers (100/300)	CHM external use at Tim-Mee acupoint + odor therapy + laser irradiation therapy.	C1: CHM external use at acupoints.C2: odor therapy.C3: laser therapy.	Not reported	1	No
Gu et al.^37^ 2012	Adult smokers (800/100)	CHM external use at Tim-Mee, LU7 acupoints + psychological counseling.	Psychological counseling.	2 w	1	No
Sun et al.^38^ 2016	Adult smokers (30/30)	CHM external use at Tim-Mee acupoint + varenicline.	Varenicline	2 w	7	No
Zhao et al.^39^ 2018	Adult smokers (124/76)	CHM external use at Tim-Mee acupoint + smoking cessation education.	Placebo CHM external use at Tim-Mee acupoint.	4 w	2	No
Zheng et al.^40^ 2019	Adult smokers (8/27)	CHM external use at body acupoints + 5As counseling.	Nicotine patch daily + 5As counseling.	6 w	1, 3, 4, 5, 7	No
Liu et al.^34^ 2019	Adult smokers I/C1/C2 (120/120/120)	CHM external use at body acupoints + placebo nicotine patch.	C1: CHM external use + nicotine patch. C2: Placebo CHM + placebo nicotine patch.	4 w	1, 3, 5, 7	No
**Auricular acupressure**						
Ma et al.^41^ 2014	Adult smokers (68/68)	Auricular acupressure at body and auricular acupoints.	Sham acupuncture	8 w	1	No
Guo et al.^42^ 2020	Adult smokers with angina pectoris (60/60)	Auricular acupressure + 5As counseling.	No treatment	4 w	1	No
**Filiform needle acupuncture alone or combined with auricular acupressure**						
Ji et al.^43^ 2023	Adult smokers diagnosed with heavy nicotine dependence (32/32)	Filiform needle acupuncture combined with auricular acupressure at body and auricular acupoints.	Nicotine patches	8 w	1, 2, 3, 5	Yes
Wang et al.^44^ 2018	Adult smokers I1/I2/C (100/100/100)	I1: filiform needle acupuncture at body acupoints. I2: auricular acupressure at auricular acupoints.	Nicotine patches	8 w	1, 3, 5	Yes
Jang et al.^45^ 2019	Adult smokers (20/21)	Acupuncture at body and auricular acupoints + aromatherapy + nicotine patches + counseling.	Nicotine patches	4 w	1, 2, 3, 5, 6	Yes
She et al.^46^ 2021	Adult smokers (30/30)	Acupuncture + auricular acupressure at body and auricular acupoints.	Nicotine patches	8 w	1, 3, 4, 5	No
Zhang et al.^47^ 2022	Adult smokers (41/41)	Acupuncture + auricular acupressure at body and auricular acupoints.	Nicotine patches	8 w	1, 2, 4	No
Chen et al.^48^ 2022	Adult smokers (100/100)	Auricular acupressure + TEAS at body and auricular acupoints.	Nicotine patches	8 w	1, 3, 5	Yes
**Fire needle acupuncture**						
He et al.^49^ 2019	Adult smokers (30/30)	Fire needle acupuncture at body acupoints.	Smoking cessation education	3 w	1, 2, 4	Yes
**TEAS**						
Lambert et al.^50^ 2011	Adult smokers I1/C1 (20/20) I2/C2/C3 (21/20/17)	TEAS (10 mA) at body acupoints.	Placebo TEAS (5 mA or 0 mA)	4 w	4, 6	No
Bilici et al.^51^ 2016	Adult smokers (84/80)	TEAS at auricular acupoints.	Sham TEAS	4 w	1, 2, 5	Yes
Li et al.^52^ 2020	Adult smokers (31/31)	TEAS at body acupoints.	Varenicline	4 w	3, 5	No
**Laser acupuncture**						
Velangi et al.^53^ 2021	Adult smokers I1/I2/C (50/50/50)	I1: auricular laser acupuncture. I2: laser acupuncture + counseling.	Counseling	4 w	5	Yes
Yavagal et al.^54^ 2021	Adult smokers I1/I2/C (20/20/20)	I1: auricular laser acupuncture. I2: auricular laser acupuncture + counseling.	Psychological counseling	4 w	5	Yes

TEAS: transcutaneous electrical acupoint stimulation. CHM: Chinese herbal medicine. NRT: nicotine replacement treatment. w: weeks. Outcomes: 1. Abstinence rate, 2. Daily cigarette consumption, 3. Withdrawal symptoms, 4. Level of exhaled CO, 5. nicotine dependence, 6. Craving for cigarettes, and 7. Relapse rate.

### Quality assessment

**Figure 2:**
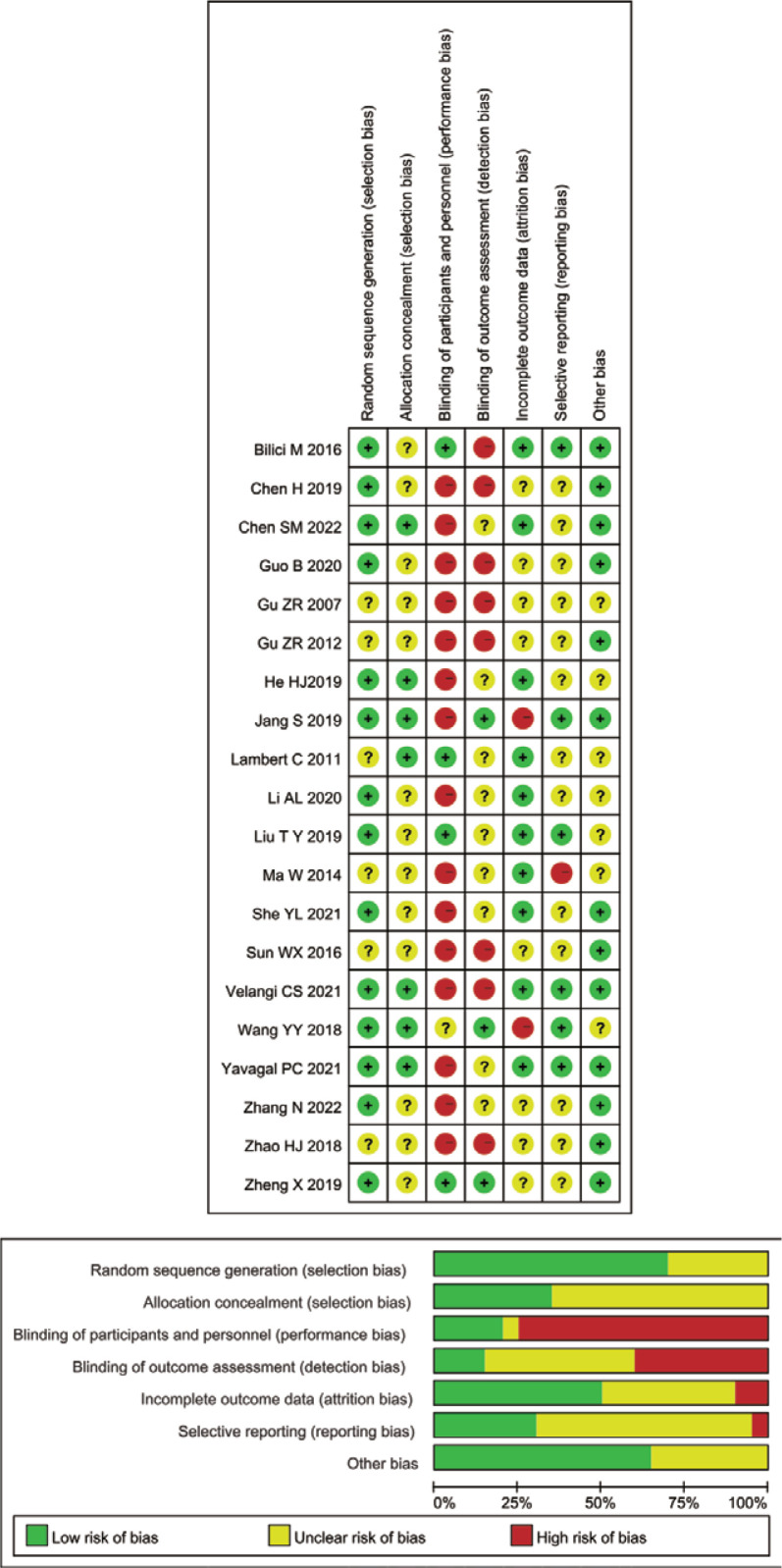
Risk of bias summary of an umbrella review and updated meta-analysis about acupuncture and related acupoint therapies for smoking cessation in adult cigarette smokers

Regarding the methodological quality for 13 SRs^10,17-19,27-35^ by AMSTAR-2, eleven (85%) SRs were of critically low quality due to either the lack of a study protocol, the list of excluded studies, or inadequate details of included studies. One Cochrane Systematic Review^10^ was assessed as moderate quality since there was more than one noncritical weakness. A single review^19^ was rated low quality due to one critical flaw. The details are presented in Table 1. We did not reassess the risk of bias of the 33 RCTs from the included SRs since they had been evaluated in the SRs. In terms of 20 unevaluated RCTs, random sequence generation was not reported in detail in 6 (30%) RCTs, and allocation concealment was not fully described in 13 (65%) RCTs. Participants and personnel were not blinded in 16 (80%) RCTs, and therefore, were assessed as high risk of performance bias. Outcome assessment was blinded in only 3 (15%) RCTs and were of low risk of bias. Attrition bias, reporting bias, and other biases were also assessed. The details of the risk of bias are shown in Figure 2.

### Effects of interventions

*Findings from 13 systematic reviews*

One systematic review^17^ suggested that various acupuncture techniques were not superior to sham acupuncture in improving abstinence rate (OR=1.20; 95% CI: 0.98–1.48) at the end of treatment or at follow-up at six months (OR=1.29; 95% CI: 0.82–2.01). However, an updated Cochrane Review^10^ indicated that acupuncture was more effective than sham acupuncture for short-term smoking cessation (RR=1.22; 95% CI: 1.08–1.38) but failed to show a long-term effect (RR=1.10; 95% CI: 0.86–1.40), the findings were consistent with other five reviews^18,27,28,31,35^. Compared with NRT, acupuncture was inferior to NRT in short-term or long-term smoking cessation (RR=0.96; 95% CI: 0.77–1.18)^35^, this finding was consistent with other three reviews^10,18,29^. Liu TY^34^ also found that filiform needle acupuncture combined with auricular acupressure was less effective than NRT for smoking cessation (OR=0.85; 95% CI: 0.36–2.03). Regarding auricular acupressure, acupressure was found more effective than sham acupressure in improving short-term abstinence rate (RR=2.54; 95% CI: 1.27–5.08)^10^, which was consistent with other four reviews^17,19,29,32^. Additionally, Kim^33^ also found that the abstinence rate was significantly higher with auricular acupressure versus simple advice (OR=22.2; 95% CI: 5.0–99.3) at follow-up at 12 months. Another systematic review^19^ included various non-traditional filiform needle acupuncture therapies and found that acupoint catgut embedding may be comparable to bupropion for short-term smoking cessation (RR=0.99; 95% CI: 0.70–1.40). Apart from the above-mentioned, acupuncture therapies, laser acupuncture, TEAS, and intradermal needle acupuncture were also evaluated.

### Updated meta-analysis classified by different acupuncture and related acupoint therapies based on all available RCTs

We only pooled the data on abstinence rate to make the results clear and direct. Two time periods were employed to report the outcome of abstinence rate to evaluate the therapeutic effects of acupuncture and related acupoint therapies in short-term (≤6 months) and long-term (>6 months), when there were sufficient data. GRADE approaches were employed to assess the certainty of updated evidence (Table 3).

**Table 3:** Evidence summary of smoking cessation: acupuncture and related acupoint therapies versus sham acupuncture or conventional therapy

Certainty assessment							Number of patients		Effect		Certainty GRADE
Number of studies	Study design	Risk of bias	Inconsistency	Indirectness	Imprecision	Publication bias	Acupuncture n/N (%)	Control n/N (%)	Relative RR (95% CI)	Absolute (95% CI)	
**1. Filiform needle acupuncture vs sham acupuncture/conventional therapy**											
**Short-term abstinence rate (filiform needle acupuncture vs sham acupuncture)**											
9	RCTs	serious^a^	serious^c^	not serious	not serious	undetected	244/717 (34.0)	166/641 (25.9)	1.44 (1.02–2.02)	114 more per 1000 (from 5 more to 264 more)	⊕⊕◯◯ Low
**Long-term abstinence rate (filiform needle acupuncture vs sham acupuncture)**											
6	RCTs	serious^a^	not serious	not serious	serious^b^	undetected	77/517 (14.9)	64/458 (14.0)	1.01 (0.63–1.61)	1 more per 1000 (from 52 fewer to 85 more)	⊕⊕◯◯ Low
**Short-term abstinence rate (filiform needle acupuncture vs NRT)**											
2	RCTs	serious^a^	not serious	not serious	serious^b^	undetected	79/372 (21.2)	89/313 (28.4)	0.76 (0.59–0.99)	68 fewer per 1000 (from 117 fewer to 3 fewer)	⊕⊕◯◯ Low
**2. Filiform needle acupuncture + auricular acupressure vs nicotine patches**											
**Short-term abstinence rate**											
6	RCTs	serious^a^	not serious	not serious	serious^b^	undetected	67/261 (25.7)	68/263 (25.9)	0.99 (0.74–1.32)	3 fewer per 1000 (from 67 fewer to 83 more)	⊕⊕◯◯ Low
**3. Acupressure vs sham acupressure/conventional therapy**											
**Short-term abstinence rate (acupressure vs conventional therapy)**											
8	RCTs	serious^a^	not serious	not serious	serious^b^	undetected	102/303 (33.7)	68/292 (23.3)	1.46 (1.14–1.87)	107 more per 1000 (from 33 fewer to 203 more)	⊕⊕◯◯ Low
**Short-term abstinence rate (acupressure vs sham acupressure)**											
2	RCTs	serious^a^	not serious	not serious	serious^b^	undetected	20/108 (18.5)	8/102 (7.8)	2.44 (1.13–5.25)	113 more per 1000 (from 10 more to 333 more)	⊕⊕◯◯ Low
**Long-term abstinence rate (acupressure vs sham acupressure)**											
2	RCTs	not serious	not serious	not serious	serious^b^	undetected	7/36 (19.4)	4/38 (10.5)	1.85 (0.59–5.82)	89 more per 1000 (from 43 fewer to 507 more)	⊕⊕⊕◯ Moderate
**4. Intradermal needle vs sham intradermal needle/counseling**											
**Short-term abstinence rate (intradermal needle vs counseling)**											
3	RCTs	not serious	not serious	not serious	serious^b^	undetected	28/81 (34.6)	26/84 (31.0)	1.12 (0.72–1.73)	37 more per 1000 (from 87 fewer to 226 more)	⊕⊕⊕◯ Moderate
**Short-term abstinence rate (intradermal needle vs sham intradermal needle)**											
2	RCTs	serious^a^	serious^c^	not serious	serious^b^	undetected	38/91 (41.8)	14/90 (15.6)	3.49 (0.40–30.59)	387 more per 1000 (from 93 fewer to 1000 more)	⊕◯◯◯ Very low
**5. TEAS vs sham TEAS**											
**Short-term abstinence rate**											
4	RCTs	not serious	not serious	not serious	serious^b^	undetected	28/145 (19.3)	20/140 (14.3)	1.33 (0.79–2.24)	47 more per 1000 (from 30 fewer to 177 more)	⊕⊕⊕◯ Moderate
**Long-term abstinence rate**											
1	RCTs	not serious	not serious	not serious	serious^b^	undetected	1/38 (2.6)	2/38 (5.3)	0.50 (0.05–5.28)	26 fewer per 1000 (from 50 fewer to 225 more)	⊕⊕⊕◯ Moderate
**6. Laser acupuncture vs sham laser acupuncture**											
**Short-term abstinence rate**											
2	RCTs	not serious	serious^c^	not serious	serious^b^	undetected	97/231 (42.0)	33/196 (16.8)	2.98 (0.24–37.81)	333 more per 1000 (from 128 fewer to 1000 more)	⊕⊕◯◯ Low
**Long-term abstinence rate**											
2	RCTs	not serious	not serious	not serious	serious^b^	undetected	26/75 (34.7)	12/85 (14.1)	2.25 (1.23–4.11)	176 more per 1000 (from 32 more to 439 more)	⊕⊕⊕◯ Moderate
**7. Acupoint catgut embedding vs bupropion/varenicline**											
**Short-term abstinence rate**											
2	RCTs	serious^a^	not serious	not serious	serious^b^	undetected	37/89 (41.6)	37/88 (42.0)	0.99 (0.70–1.40)	4 fewer per 1000 (from 126 fewer to 168 more)	⊕⊕◯◯ Low
**8. External use of Chinese herbal medicine at acupoint vs conventional therapy/placebo**											
**Short-term abstinence rate (external use of Chinese herbal medicine vs conventional therapy)**											
2	RCTs	serious^a^	serious^c^	not serious	not serious	undetected	677/828 (81.8)	28/127 (22.0)	2.35 (0.43–12.93)	276 more per 1000 (from 126 fewer to 1000 more)	⊕⊕◯◯ Low

RR: risk ratio.

a Blinding method was not used.

b Number of events was small.

c I^2^ was large.

### Filiform needle acupuncture versus sham acupuncture/NRT

**Figure 3:**
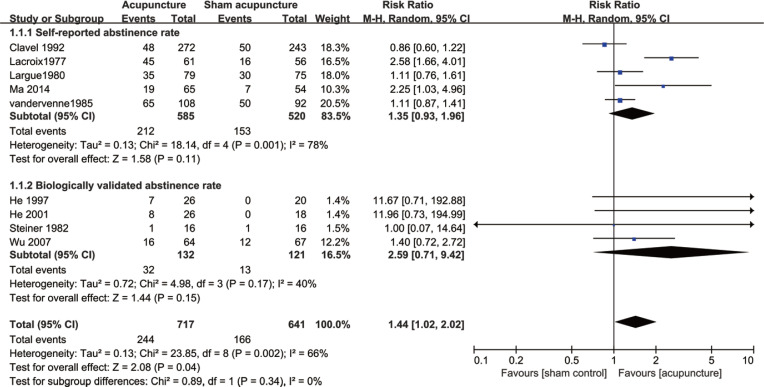
Filiform needle acupuncture vs sham acupuncture for short-term smoking cessation

We found that traditional filiform needle acupuncture was more effective than sham acupuncture in achieving short-term smoking cessation (RR=1.44; 95% CI: 1.02–2.02; I^2^= 66%; low certainty; 9 RCTs, n=1358) (Figure 3, Table 3). However, subgroup analysis by means of abstinence rate measures suggested that filiform needle acupuncture was not superior to sham acupuncture in improving short-term self-reported abstinence rate (RR=1.35; 95% CI: 0.93–1.96; I^2^=78%; 5 RCTs, n=1105) or biologically validated abstinence rate (RR=2.59; 95% CI: 0.71–9.42; I^2^=40%; 4 RCTs, n=253) (Figure 3).

Traditional filiform needle acupuncture also failed to show a better effect than sham acupuncture in improving long-term abstinence rate (RR=1.01; 95% CI: 0.63–1.61; I^2^=34%; low certainty; 6 RCTs, n=975) (Supplementary file Figure 2, Table 3). Compared with NRT, the result suggested that filiform needle acupuncture was less effective than NRT in achieving short-term smoking cessation (RR=0.76; 95% CI: 0.59–0.99; low certainty; 2 RCTs, n=685) (Table 3).

*Filiform needle acupuncture + auricular acupressure versus nicotine patches*

**Figure 4:**
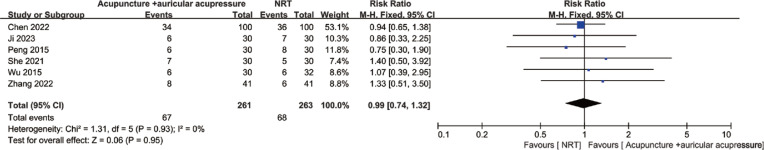
Filiform needle acupuncture + auricular acupressure vs nicotine patches for short-term smoking cessation

Six RCTs compared the effect of filiform needle acupuncture combined with auricular acupressure and nicotine patches. The pooled data suggested that there was no significant difference between the acupuncture group and the nicotine patch group (RR=0.99; 95% CI: 0.74–1.32; low certainty; 6 RCTs, n=524) (Figure 4, Table 3).

*Auricular acupressure versus sham acupressure/counseling*

Ten RCTs involving 905 participants reported the short-term abstinence rate. The pooled data suggested that auricular acupressure was superior to both sham acupressure (RR=2.44; 95% CI: 1.13– 5.25; low certainty; RCTs, n=210) and conventional therapy (RR=1.46; 95% CI: 1.14–1.87; I^2^=5%; low certainty; 8 RCTs, n=595) in achieving short-term smoking cessation (Supplementary file Figure 3, Table 3). Conventional therapy mainly refers to behavioral counseling. However, the long-term abstinence effect was not significant between acupressure and sham acupressure (RR=1.85; 95% CI: 0.59–5.82; I^2^=14%; moderate certainty; 2 RCTs, n=74) (Table 3). A funnel plot shows asymmetry, with larger studies typically showing smaller effects and an absence of small negative studies (Supplementary file Figure 4).

*Intradermal needle versus sham intradermal needle/counseling*

Five RCTs on intradermal needles reported a short-term abstinence rate involving 346 participants. We found that intradermal needle failed to show a better effect than counseling (RR=1.12; 95% CI: 0.72–1.73; moderate certainty; 3RCTs, n=165) or sham intradermal needle (RR=3.49; 95% CI: 0.40–30.59; I^2^=88%; very low certainty; 2 RCTs, n=181) (Table 3) in achieving short-term smoking cessation.

*TEAS versus sham TEAS*

Compared with sham TEAS, TEAS failed to show a beneficial effect in achieving short-term (RR=1.33; 95% CI: 0.79–2.24; moderate certainty; 4 RCTs, n=285) and long-term (RR=0.50; 95% CI: 0.05–5.28; moderate certainty; 1 RCT, n=76) smoking cessation (Table 3).

*Laser acupuncture versus sham laser acupuncture*

Laser acupuncture failed to show a better effect than sham laser acupuncture in improving short-term abstinence rate (RR=2.98; 95% CI: 0.24–37.81; I^2^=96%; low certainty; 2 RCTs, n=427) (Table 3). However, laser acupuncture appeared to be more effective in achieving long-term smoking cessation (RR=2.25; 95% CI: 1.23–4.11; moderate certainty; 2 RCTs, n=160) (Table 3). However, the included 2 RCTs^53,54^ failed to report the outcome of the abstinence rate; therefore, we were unable to pool the data.

*Acupoint catgut embedding versus bupropion/varenicline*

Absorbable catgut embedding was a more recently developed acupuncture technique providing continuous stimulation. Two RCTs included in one systematic review^19^ compared the effect of acupoint catgut embedding with bupropion or varenicline for smoking cessation and found that acupoint catgut embedding was comparable to medication in improving short-term abstinence rate (RR=0.99; 95% CI: 0.70–1.40; low certainty; 2RCTs, n=177) (Table 3).

*External use of Chinese herbal medicine at acupoint versus conventional therapy/placebo*

A total of 6 RCTs on Chinese herbal medicine of external use at acupoints were identified in this update. However, due to unreported abstinence rates or control variation, the data from only 2 RCTs were pooled together. The result suggested that Chinese herbal medicine external use at acupoints combined with conventional therapy was not superior to conventional therapy alone in achieving short-term smoking cessation (RR=2.35; 95% CI: 0.43–12.93; low certainty; 2 RCTs, n=955) (Table 3).

*Fire needle*

Only one RCT on fire needle acupuncture for smoking cessation was identified. The result suggested that fire needle acupuncture had more benefits than behavioral counseling in achieving short-term smoking cessation (RR=1.82; 95% CI: 1.07–3.10; 1 RCT, n=60), but the long-term effect (RR=1.47; 95% CI: 0.97–2.23; 1 RCT, n=60) was not observed.

### Adverse events

The majority of SRs and RCTs on acupuncture and related therapies failed to report any serious adverse events. Infrequent minor bleeding or bruising upon needle removal was reported in RCTs on filiform needle acupuncture. Transient and minor adverse events, including itching, mild tenderness, and feeling hot, were reported in RCTs on auricular acupressure. Mild pain, soreness, and minor swelling in 2 cases were reported in RCTs on acupoint catgut embedding. All the adverse events reported were mild and relieved after the removal of acupuncture. No serious adverse events were reported in the included SRs and RCTs.

### Certainty of evidence

GRADE approach was employed to evaluate the certainty of updated evidence. The certainty of the evidence was downgraded to low or very low certainty due to the risk of bias, imprecision, inconsistency or indirectness. The detailed evidence summary of the abstinence rate is presented in Table 3.

## DISCUSSION

### Findings from systematic reviews

A total of 13 SRs and 20 RCTs were identified in this umbrella review. All the included SRs were based on RCTs or quasi-RCTs, and the methodological quality for 85% of SRs was critically low, assessed by AMSTAR-2. We classified these SRs into different categories according to acupuncture techniques used, and we found that only 3 SRs^10,17,29^ covered a more comprehensive range of acupuncture techniques, such as body filiform needle acupuncture, auricular acupressure, TEAS, intradermal needle, or laser acupuncture. However, RCTs on acupoint catgut embedding or Chinese herbal medicine external use at acupoints for smoking cessation were still lacking in these 3 SRs. The remaining SRs focused on auricular acupuncture, body filiform needle acupuncture, or non-traditional filiform needle acupuncture therapies separately. Six reviews^10,18,27,28,31,35^ consistently found that body filiform needle acupuncture was more effective than sham acupuncture for short-term smoking cessation, but this effect was not observed for long-term smoking cessation. However, acupuncture was inferior to NRT for quitting smoking^10,18,29,35^, and the findings were similar with auricular acupressure. Therefore, we aimed to provide comprehensive and updated evidence based on various acupuncture and related acupoint therapies for smoking cessation.

### Findings from an updated meta-analysis

Twenty RCTs involving 3532 participants were identified, and about 80% of RCTs were of high risk of performance bias due to the absence of blinding of participants and personnel. GRADE approaches were employed to assess the certainty of evidence. In terms of filiform acupuncture, low certainty evidence suggested that body filiform needle acupuncture was superior to sham acupuncture in achieving short-term smoking cessation. Still, this effect was not observed for long-term smoking cessation. Compared with NRT, filiform needle acupuncture was less effective than NRT in achieving short-term smoking cessation. However, low certainty evidence suggested that body filiform needle acupuncture combined with auricular acupressure may be comparable to NRT patches in achieving short-term smoking cessation. In terms of auricular acupressure, low certainty evidence suggested that acupressure was superior to sham acupressure in achieving short-term smoking cessation. Acupressure also showed a more beneficial effect than counseling in achieving short-term smoking cessation. In terms of the intradermal needle, TEAS, and laser therapy, low to moderate evidence suggested that these therapies failed to show a better effect than sham control or counseling for short-term smoking cessation. In terms of acupoint catgut embedding, low certainty evidence suggested that it was potentially comparable to bupropion/varenicline in improving the short-term abstinence rate. External use of Chinese herbal medicine at acupoints in combination with conventional therapy failed to show a better effect than conventional therapy alone in achieving short-term smoking cessation.

### Comparisons with other studies

This was an umbrella review and updated meta-analysis based on comprehensive acupuncture and related acupoint therapies. The previous similar SRs^10,17,27-29^ had not included various acupuncture and related acupoint stimulation therapies, such as acupoint catgut embedding, fire needle, or Chinese herbal medicine external use at acupoints. Additionally, the data were not pooled based on different acupuncture techniques in many previous SRs. A latest umbrella review^55^ on traditional Chinese medicine for smoking cessation also included RCTs on acupuncture techniques; however, the data were not pooled based on different acupuncture therapies but were pooled together. Outcomes of smoking cessation from different acupuncture therapies may result in large heterogeneity if pooled together. Therefore, this umbrella review separated acupuncture therapies into more specific interventions, such as traditional filiform acupuncture, TEAS, intradermal acupuncture, acupressure, laser acupuncture, and acupoint catgut embedding. Therefore, the data were pooled based on different acupuncture techniques. GRADE approaches were employed to evaluate the certainty of the updated evidence. We focused on the most important outcome-abstinence rate and aimed to provide an evidence profile of various acupuncture therapies for smoking cessation.

### Strengths and limitations

This umbrella review summarized the current evidence about the effectiveness of various acupuncture and related acupoint therapies for smoking cessation. We classified the results according to different types of acupuncture therapies and assessed the certainty of evidence with the GRADE approach. However, this study has some limitations. Firstly, we only focused on the outcome of abstinence rate in order to focus on the results; other outcomes such as withdrawal symptoms, nicotine dependence, and relapse rate should also be evaluated in the future. The abstinence rate was usually self-reported and there was a lack of biochemical validation. Additionally, conventional cigarette smoking was only considered in this study. Secondly, substantial clinical or methodological heterogeneity among studies hampers data synthesis. Lastly, the certainty of evidence was downgraded to moderate or low quality due to lack of blinding method in primary studies (risk of bias), small number of events (imprecision), or large I^2^ value (inconsistency). Therefore, the results should be interpreted with caution. However, a comprehensive evidence picture of acupuncture and related acupoint therapies for smoking was provided in this umbrella review.

### Implications

For clinical practice, although we found that body needle acupuncture was effective for smoking cessation, an interview study^56^ indicated that patient’s fear of pain from body needle acupuncture, and the inconvenience of attending appointments may reduce treatment compliance. Smoking cessation requires long-term adherence to treatment, and therefore, auricular acupressure or other self-administered therapies may be more appropriate in clinical practice. Regarding study design, there is a need to improve the certainty of evidence to inform decision-making for smoking cessation. The blinding method should be applied to decrease performance bias, consistency in acupuncture interventions, and the report of outcomes, which needs to be improved in future trials.

## CONCLUSIONS

The current low certainty evidence suggests that body filiform needle acupuncture, auricular acupressure, and acupoint catgut embedding appear to be safe and effective in achieving short-term smoking cessation. Body filiform needle acupuncture combined with auricular acupressure may be comparable to NRT in achieving short-term smoking cessation. However, more rigorously designed RCTs with larger sample size, biologically validated abstinence rate, and long-term follow-up data are warranted to further verify these effects.

## Supplementary Material



## Data Availability

Data sharing is not applicable to this article as no new data were created.

## References

[1] GBD 2019 Risk Factors Collaborators. Global burden of 87 risk factors in 204 countries and territories, 1990-2019: a systematic analysis for the Global Burden of Disease Study 2019. Lancet. 2020;396(10258):1223-1249. doi:10.1016/S0140-6736(20)30752-2 33069327 PMC7566194

[2] World Health Organization. Tobacco: Overview. World Health Organization. Accessed March 15, 2024. https://www.who.int/health-topics/tobacco#tab=tab_1

[3] Chan KH, Wright N, Xiao D, et al. Tobacco smoking and risks of more than 470 diseases in China: a prospective cohort study. Lancet Public Health. 2022;7(12):e1014-e1026. doi:10.1016/S2468-2667(22)00227-4 36462513 PMC7613927

[4] National Health Commission of the People’s Republic of China. The National Health Commission released the “China Smoking Hazards to Health Report 2020”. In Chinese. May 28, 2021. Accessed March 15, 2024. http://www.nhc.gov.cn/guihuaxxs/s7788/202105/c1c6d17275d94de5a349e379bd755bf1

[5] Shields PG, Bierut L, Arenberg D, et al. Smoking Cessation, Version 3.2022, NCCN Clinical Practice Guidelines in Oncology. J Natl Compr Canc Netw. 2023;21(3):297-322. doi:10.6004/jnccn.2023.0013 36898367

[6] The People's Republic of China National Health Committee. Chinese Clinical Guidelines for Smoking Cessation; 2015.

[7] Zhao X. Analysis of the construction status of some smoking cessation clinics in China in 2016. Chinese Center Dis. Control Prevent; 2018.

[8] Aumann I, Rozanski K, Damm K, Graf von der Schulenburg JM. Kosteneffektivität von pharmakologischen Raucherentwöhnungsmaßnahmen – ein systematisches Literaturreview. Gesundheitswesen. 2016;78(10):660-671. doi:10.1055/s-0035-1548852 27784123

[9] Gilbey V, Neumann B. Auricular acupuncture for smoking withdrawal. Am J Acupunct. 1977;(5):239–247.

[10] White AR, Rampes H, Liu JP, Stead LF, Campbell J. Acupuncture and related interventions for smoking cessation. Cochrane Database Syst Rev. 2014;2014(1):CD000009. doi:10.1002/14651858.CD000009.pub4 24459016 PMC7263424

[11] Qiao L, Guo M, Qian J, Xu B, Gu C, Yang Y. Research advances on acupuncture analgesia. Am J Chin Med. 2020;48(2):245-258. doi:10.1142/S0192415X20500135 32138535

[12] Wang YY, Liu Z, Chen F, et al. Effects of acupuncture on craving after tobacco cessation: a resting-state fMRI study based on the fractional amplitude of low-frequency fluctuation. Quant Imaging Med Surg. 2019;9(6):1118-1125. doi:10.21037/qims.2019.06.07 31367566 PMC6629569

[13] Lee EJ. The effect of auricular acupressure and positive group psychotherapy with motivational interviewing for smoking cessation in Korean adults. Holist Nurs Pract. 2020;34(2):113-120. doi:10.1097/HNP.0000000000000348 31567305

[14] Fritz DJ, Carney RM, Steinmeyer B, Ditson G, Hill N, Zee-Cheng J. The efficacy of auriculotherapy for smoking cessation: a randomized, placebo-controlled trial. J Am Board Fam Med. 2013;26(1):61-70. doi:10.3122/jabfm.2013.01.120157 23288282

[15] Yiming C, Changxin Z, Ung WS, Lei Z, Kean LS. Laser acupuncture for adolescent smokers--a randomized double-blind controlled trial. Am J Chin Med. 2000;28(3-4):443-449. doi:10.1142/S0192415X00000520 11154059

[16] Li HY, He W. Comparative study of acupoint catgut embedding and bupropion hydrochloride sustained-release tablets for tobacco dependence. Article in Chinese. Zhongguo Zhen Jiu. 2019;39(4):384-388. doi:10.13703/j.0255-2930.2019.04.010 30957449

[17] White AR, Resch KL, Ernst E. A meta-analysis of acupuncture techniques for smoking cessation. Tob Control. 1999;8(4):393-397. doi:10.1136/tc.8.4.393 10629245 PMC1759757

[18] Wang JH, van Haselen R, Wang M, et al. Acupuncture for smoking cessation: a systematic review and meta-analysis of 24 randomized controlled trials. Tob Induc Dis. 2019;17(June):48. doi:10.18332/tid/109195 31516491 PMC6662782

[19] Zhang YY, Yu ZY, Lan HD, et al. Non-traditional acupuncture therapies for smoking cessation: a systematic review of randomized controlled trials. Eur J Integr Med. 2021;47:101390. doi:10.1016/j.eujim.2021.101390

[20] Aromataris E, Fernandez R, Godfrey CM, Holly C, Khalil H, Tungpunkom P. Summarizing systematic reviews: methodological development, conduct and reporting of an umbrella review approach. Int J Evid Based Healthc. 2015;13(3):132-140. doi:10.1097/XEB.0000000000000055 26360830

[21] Pollock M, Fernandes RM, Becker LA, Becker LA, Pieper D, Hartling L. Chapter V: Overviews of Reviews. In: Higgins J, Thomas J, Chandler J, et al. Cochrane Handbook for Systematic Reviews of Interventions: Version 6.4, 2023. Cochrane; 2023. Accessed March 15, 2024. https://training.cochrane.org/handbook

[22] Page MJ, McKenzie JE, Bossuyt PM, et al. The PRISMA 2020 statement: an updated guideline for reporting systematic reviews. J Clin Epidemiol. 2021;134:178-189. doi:10.1016/j.jclinepi.2021.03.001 33789819

[23] Zhang YY, Su YZ, Tian ZY, et al. Acupuncture and related acupoint therapies for smoking cessation: a protocol of an overview and updated meta-analysis. INPLASY protocol 202410106. Preprint posted online January 25, 2024. doi:10.37766/inplasy2024.1.0106 PMC11025526 38638420

[24] Shea BJ, Reeves BC, Wells G, et al. AMSTAR 2: a critical appraisal tool for systematic reviews that include randomised or non-randomised studies of healthcare interventions, or both. BMJ. 2017;358:j4008. doi:10.1136/bmj.j4008 28935701 PMC5833365

[25] Higgins J, Thomas J, Chandler J, et al. Cochrane Handbook for Systematic Reviews of Interventions. Version 6.1, 2020. Cochrane; 2020. Accessed March 15, 2024. https://training.cochrane.org/handbook/archive/v6.1

[26] Guyatt GH, Oxman AD, Vist GE, et al. GRADE: an emerging consensus on rating quality of evidence and strength of recommendations. BMJ. 2008;336(7650):924-926. doi:10.1136/bmj.39489.470347.AD 18436948 PMC2335261

[27] Cheng HM, Chung YC, Chen HH, Chang YH, Yeh ML. Systematic review and meta-analysis of the effects of acupoint stimulation on smoking cessation. Am J Chin Med. 2012;40(3):429-442. doi:10.1142/S0192415X12500334 22745061

[28] Liu Z, Wang Y, Wu Y, Yang J. Condition and effectiveness evaluation of acupuncture for smoking cessation. Article in Chinese. Chinese Acupuncture & Moxibustion. 2015; 35(8):851-857. 26571912

[29] Dai R, Cao Y, Zhang H, et al. Comparison between acupuncture and nicotine replacement therapies for smoking cessation based on randomized controlled trials: a systematic review and Bayesian Network meta-analysis. Evid Based Complement Alternat Med. 2021;2021:9997516. doi:10.1155/2021/9997516 34221095 PMC8225439

[30] White A, Moody R. The effects of auricular acupuncture on smoking cessation may not depend on the point chosen--an exploratory meta-analysis. Acupunct Med. 2006;24(4):149-156. doi:10.1136/aim.24.4.149 17264832

[31] Tahiri M, Mottillo S, Joseph L, Pilote L, Eisenberg MJ. Alternative smoking cessation aids: a meta-analysis of randomized controlled trials. Am J Med. 2012;125(6):576-584. doi:10.1016/j.amjmed.2011.09.028 22502956

[32] Di YM, May BH, Zhang AL, Zhou IW, Worsnop C, Xue CC. A meta-analysis of ear-acupuncture, ear-acupressure and auriculotherapy for cigarette smoking cessation. Drug Alcohol Depend. 2014;142:14-23. doi:10.1016/j.drugalcdep.2014.07.002 25064021

[33] Kim SS, Chen W, Kolodziej M, Wang X, Wang VJ, Ziedonis D. A systematic review of smoking cessation intervention studies in China. Nicotine Tob Res. 2012;14(8):891-899. doi:10.1093/ntr/ntr289 22249687

[34] Liu TY. Study on the clinical efficacy and safety evaluation of Chinese traditional medicine Babu patch for smoking cessation in interfering with tobacco dependence (phlegm and blood stasis syndrome). In Chinese. Dissertation. Liaoning University of Traditional Chinese Medicine; 2019. doi:10.27213/d.cnki.glnzc.2019.000021

[35] Kuang HJ, Tang J, Fu SS, Cao Y, Zhong F. Systematic Evaluation of Acupuncture and Moxibustion for the Treatment of Tobacco Dependence. ACTA Chinese medicine. 2022;37(3):661-672. doi:10.16368/j.issn.1674-8999.2022.03.124

[36] Gu ZR. Clinical study of 400 smokers treated by acupoint stimulation. The 13th National Symposium on Smoking and the third Framework Convention on Tobacco Control; 2007. doi:10.26914/c.cnkihy.2018.006802

[37] Gu ZR. Summary of 900 Cases of Smoking Cessation Treated with Chinese Medicinal Plaster and Psychological Counseling. World Journal of Integrated Traditional and Western Medicine. 2012;7(8):703-705. doi:10.13935/j.cnki.sjzx.2012.08.032

[38] Sun WX. Effect of Chinese herbal smoking cessation patches on the relapse rate of nicotine dependence. Guangming Journal of Chinese Medicine. 2016;31(03):456-457.

[39] Zhao HJ, Ren XL, Chang XH, et al. Study on the effect of self-made traditional Chinese herbal medicine external use at acupoint on smoking cessation. Journal of Gansu University of Chinese Medicine. 2018;35(1):77-80. doi:10.16841/j.issn1003-8450.2018.01.21

[40] Zheng X, Qu NN, Ma LJ, et al. Clinical Study on the Intervention of TCM Quit Smoking Paste Combined with “5A” Program on Tobacco Dependent Population with Syndrome of Phlegm and Blood Stasis. Journal of Liaoning University of Traditional Chinese Medicine. 2019;21(3):113-116. doi:10.13194/j.issn.1673-842x.2019.03.031

[41] Ma W, Xue JC. Effects of acupuncture and moxibustion on the contents of sialic acid and C-reactive protein in patients with smoking cessation. Journal of Modern Integrated Traditional and Western Medicine. 2014;23(30). doi:10.3969/j.issn.1008-8849.2014.30.011

[42] Guo B, Gong LL. Effect Analysis of Traditional Chinese Medicine Nursing of Auricular Acupoint Pressing Combined with “5A” Method on Smoking Cessation in Patients with Angina Pectoris. Chinese Medical Guide. 2020;18(22):153-154. doi:10.15912/j.cnki.gocm.2020.22.070

[43] Ji KS, She YL, Chen YX, et al. Acupuncture combined with auricular acupoints patches for moderate to severe nicotine dependence: a randomized controlled study. Journal of Traditional Chinese Medicine. 2023; 64(17):1771-1776. doi:10.13288/j.11-2166/r.2023.17.009

[44] Wang YY, Liu Z, Wu Y, et al. Efficacy of acupuncture Is noninferior to nicotine replacement therapy for tobacco cessation: results of a prospective, randomized, active-controlled open-label trial. Chest. 2018;153(3):680-688. doi:10.1016/j.chest.2017.11.015 29175360

[45] Jang S, Lee JA, Jang BH, Shin YC, Ko SG, Park S. Clinical effectiveness of traditional and complementary medicine interventions in combination with nicotine replacement therapy on smoking cessation: a randomized controlled pilot trial. J Altern Complement Med. 2019;25(5):526-534. doi:10.1089/acm.2019.0009 31017453

[46] She YL. Efficacy of Acupuncture Combined with Auricular Acupoints in the Intervention of Moderate and Severe Tobacco Dependence. In Chinese. Dissertation. Guangzhou University of Chinese Medicine; 2021. doi:10.27044/d.cnki.ggzzu.2021.000780

[47] Zhang N, She YL, Lin GH. Study on the clinical effect of Lieque acupuncture through Yangxi applied to patients with nicotine addiction. Doctor. 2022;7(17): 79-81. doi:10.19604/j.cnki.dys.2022.17.019

[48] Chen SM, Liu ZY, Ji J, Liu Z, Wang YY, Yang JS. Auricular point sticking combined with transcutaneous electrical acupoint stimulation for smoking cessation: a randomized controlled trial. Article in Chinese. Zhongguo Zhen Jiu. 2022;42(11):1235-1239. doi:10.13703/j.0255-2930.20220104-k0001 36397220

[49] He HJ. The Clinical Study of Fire Needle on Smoking Cessation. Dissertation. Guangzhou University of Traditional Chinese Medicine; 2019. doi:10.27044/d.cnki.ggzzu.2019.001197

[50] Lambert C, Berlin I, Lee TL, et al. A standardized transcutaneous electric acupoint stimulation for relieving tobacco urges in dependent smokers. Evid Based Complement Alternat Med. 2011;2011:195714. doi:10.1093/ecam/nen074 19073777 PMC3135870

[51] Bilici M, Güven S, Köşker S, Şafak A, Semiz ÜB. Electroacupuncture therapy in nicotine dependence: a double blind, sham-controlled study. Noro Psikiyatr Ars. 2016;53(1):28-32. doi:10.5152/npa.2015.9887 28360762 PMC5353233

[52] Li AL. The Clinical Effect of Electroacupuncture on Nicotine Dependence. Master’s Theses. Shanghai University of Chinese Medicine; 2020. doi:10.27320/d.cnki.gszyu.2020.000399

[53] Velangi CS, Yavagal PC, Nagesh L. Role of auricular laser acupuncture and psychological counseling in reducing nicotine dependence due to smoking: a randomized controlled trial. Indian J Public Health. 2021;65(3):243-249. doi:10.4103/ijph.IJPH_810_20 34558485

[54] Yavagal PC, L N. Efficacy of laser auricular acupuncture for smoking cessation: a randomised controlled trial. Sultan Qaboos Univ Med J. 2021;21(2):e275-e281. doi:10.18295/squmj.2021.21.02.017 34221476 PMC8219332

[55] Lu CL, Jin XY, Wang QY, et al. Traditional Chinese medicine for smoking cessation: an umbrella review of systematic reviews and meta-analysis of randomized controlled trials. Tob Induc Dis. 2023;21(November):150. doi:10.18332/tid/174090 38026501 PMC10647068

[56] Cao HJ, Li X, Li XL, et al. Factors influencing participant compliance in acupuncture trials: an in-depth interview study. PLoS One. 2020;15(4):e0231780. doi:10.1371/journal.pone.0231780 32298368 PMC7162473

