# Supplementary material for: Acupuncture and related acupoint therapies for smoking cessation: An umbrella review and updated meta-analysis
Source: Tob Induc Dis. 2024 Apr 18;22:10.18332/tid/186147. doi: 10.18332/tid/186147 (PMC11025526; doi:10.18332/tid/186147)
Supplement: Supplementary file 1 [file TID-22-64-s1.pdf]

Supplementary 1

Search strategy in each database

**Databases**	**Search strategy**	**Records**
Systematic review and randomized controlled trials		
**CNKI**	SU=('针刺'+'针灸'+'毫针'+'微针'+'温针'+'激光针'+'五行针'+'电针'+'火针'+'耳针'+'头针'+'体针'+'腹针'+'鼻针'+'腕踝针'+'眼针'+'穴位按压'+'穴位贴敷'+'代针膏'+'穴位注射'+'穴位刺激'+'经皮穴位电刺激'+'耳穴压豆'+'耳贴'+ '耳豆'+'皮内针'+'埋线'+'埋针') AND SU=('戒烟'+'烟草依赖'+'尼古丁依赖') AND (SU=('系统综述'+'系统评价'+'系统'+'Meta 分析'+'荟萃分析'+'汇总分析'+'集成分析'+'二次分析'+'衍生分析') OR ((FT=(随机) OR FT= (RCT)) NOT (TI=(动物) OR TI=(鼠) OR TI=(犬) OR TI=(兔) OR TI=(猴))))	89
**WanFang**	主题:("针刺"or"针灸"or"毫针"or"微针"or"温针"or"激光针"or"五行针"or"电针"or"火针"or"耳针"or"头针"or"体针"or"腹针"or"鼻针"or"腕踝针"or"眼针"or"穴位按压"or"穴位贴敷"or"代针膏"or"穴位注射"or"穴位刺激"or"经皮穴位电刺激"or"耳穴压豆"or"耳贴"or"耳豆"or"皮内针"or"埋线"or"埋针") and 主题:("戒烟"or"烟草依赖"or"尼古丁依赖") and (主题:("系统综述"or"系统评价"or"系统"or" Meta 分析"or"荟萃分析"or"汇总分析"or"集成分析"or"二次分析"or"衍生分析") or ((全部:(随机 or RCT)) not (题名:(动物 OR 鼠 OR 犬 OR 兔 OR 猴))))	83
**VIP**	(M=针刺 OR 针灸 OR 毫针 OR 微针 OR 温针 OR 激光针 OR 五行针 OR 电针 OR 火针 OR 耳针 OR 头针 OR 体针 OR 腹针 OR 鼻针 OR 腕踝针 OR 眼针 OR 穴位按压 OR 穴位贴敷 OR 代针膏 OR 穴位注射 OR 穴位刺激 OR 经皮穴位电刺激 OR 耳穴压豆 OR 耳贴 OR 耳豆 OR 皮内针 OR 埋线 OR 埋针) AND (M=戒烟 OR 烟草依赖 OR 尼古丁依赖) AND ((M=系统综述 OR 系统评价 OR 系统 OR Meta 分析 OR 荟萃分析 OR 汇总分析 OR 集成分析 OR 二次分析 OR 衍生分析) OR ((U=(随机 OR RCT)) NOT (T=(动物 OR 鼠 OR 犬 OR 兔 OR 猴))))	29
**Sino-Med**	("针刺"[常用字段:智能] OR "针灸"[常用字段:智能] OR "毫针"[常用字段:智能] OR "微针"[常用字段:智能] OR "温针"[常用字段:智能] OR "激光针"[常用字段:智能] OR "五行针"[常用字段:智能] OR "电针"[常用字段:智能] OR "火针"[常用字段:智能] OR "耳针"[常用字段:智能] OR "头针"[常用字段:智能] OR "体针"[常用字段:智能] OR "腹针"[常用字段:智能] OR "鼻针"[常用字段:智能] OR "腕踝针"[常用字段:智能] OR "眼针"[常用字段:智能] OR "穴位按	67

	压"[常用字段:智能] OR "穴位贴敷"[常用字段:智能] OR "代针膏"[常用字段:智能] OR "穴位注射"[常用字段:智能] OR "穴位刺激"[常用字段:智能] OR "经皮穴位电刺激"[常用字段:智能] OR "耳穴压豆"[常用字段:智能] OR "耳贴"[常用字段:智能] OR "耳豆"[常用字段:智能] OR "皮内针"[常用字段:智能] OR "埋线"[常用字段:智能] OR "埋针"[常用字段:智能]) AND ("戒烟"[常用字段:智能] OR "烟草依赖"[常用字段:智能] OR "尼古丁依赖"[常用字段:智能]) AND (("系统综述"[常用字段:智能] OR "系统评价"[常用字段:智能] OR "系统"[常用字段:智能] OR "Meta 分析"[常用字段:智能] OR "荟萃分析"[常用字段:智能] OR "汇总分析"OR "集成分析"[常用字段:智能] OR "二次分析"[常用字段:智能] OR "衍生分析") OR (("随机"[常用字段:智能] OR "RCT"[常用字段:智能]) NOT ("动物"[标题:智能] OR "鼠"[标题:智能] OR "犬"[标题:智能] OR "兔"[标题:智能] OR "猴"[标题:智能])))	
**PubMed**	"Medicine, Chinese Traditional"[MeSH Terms] OR "Complementary Therapies"[MeSH Terms] OR "acupuncture*"[Title/Abstract] OR "electro stimulation"[Title/Abstract] OR "electric stimulation"[Title/Abstract] OR "auricular*"[Title/Abstract] OR "laser therapy"[Title/Abstract] OR "transcutaneous"[Title/Abstract] OR "acupoint application"[Title/Abstract] OR "acupoint stimulation"[Title/Abstract] OR "auricular point sticking"[Title/Abstract] OR "acupressure*"[Title/Abstract] AND ("smoking cessation"[MeSH Terms] OR "tobacco cessation"[Title/Abstract] OR "quit smoking"[Title/Abstract] OR "stop smoking"[Title/Abstract] OR "cease smoking"[Title/Abstract] OR "nicotine withdrawal"[Title/Abstract] OR "nicotine dependence"[Title/Abstract] OR "tobacco withdrawal syndrome"[Title/Abstract]) AND (("systematic review"[Publication Type] OR "review"[Title/Abstract] OR "meta-analysis"[Title/Abstract] OR "systematic review"[Title/Abstract] OR "meta analysis"[Title/Abstract]) OR (((randomized controlled trial [Publication Type]) OR (controlled clinical trial [Publication Type]) OR (randomized [Title/Abstract]) OR (placebo [Title/Abstract]) OR (drug therapy [MeSH Subheading]) OR (randomly [Title/Abstract]) OR (trial [Title/Abstract]) OR (groups [Title/Abstract]) OR (crossover or cross-over [Text Word]) OR (pragmatic clinical trial [Publication Type])) NOT (Animals [MeSH Terms] NOT Humans [MeSH Terms])))	264

**Cochrane Library**	#1 MeSH descriptor: [Smoking Cessation] explode all trees<br>#2 (traditional Chinese medicine):ti,ab,kw<br>#3 (complementary therapy):ti,ab,kw<br>#4 (acupuncture):ti,ab,kw<br>#5 (electro stimulation):ti,ab,kw<br>#6 (electric stimulation):ti,ab,kw<br>#7 (auricular):ti,ab,kw<br>#8 (laser therapy):ti,ab,kw<br>#9 (transcutaneous):ti,ab,kw<br>#10 (acupoint application):ti,ab,kw<br>#11 (acupoint stimulation):ti,ab,kw<br>#12 (auricular point sticking):ti,ab,kw<br>#13 (acupressure):ti,ab,kw<br>#14 #2 OR #3 OR #4 OR #5 OR #6 OR #7 OR #8 OR #9 OR #10 OR #11 OR #12 OR #13<br>#15 #1 AND #14	59
**EMBASE**	#1 'traditional Chinese medicine':ab,ti OR 'complementary therapy':ab,ti OR 'acupuncture':ab,ti OR 'electro stimulation':ab,ti OR 'electric stimulation':ab,ti OR 'auricular':ab,ti OR 'laser therapy':ab,ti OR 'transcutaneous':ab,ti OR 'acupoint application':ab,ti OR 'acupoint stimulation':ab,ti OR 'auricular point sticking':ab,ti OR 'acupressure':ab,ti<br>#2 'smoking cessation':ab,ti OR 'tobacco cessation':ab,ti OR 'quit smoking':ab,ti OR 'stop smoking':ab,ti OR 'cease smoking':ab,ti OR 'nicotine withdrawal':ab,ti OR 'nicotine dependence':ab,ti OR 'tobacco withdrawal syndrome':ab,ti<br>#3 'systematic review'/exp OR 'review':ab,ti OR 'meta-analysis'/exp OR 'systematic review':ab,ti OR 'meta-analysis':ab,ti OR 'meta analysis':ab,ti<br>#4 ('crossover procedure':de OR 'double-blind procedure':de OR 'randomized controlled trial':de OR 'single-blind procedure':de OR (random* OR factorial* OR crossover* OR cross NEXT/1 over* OR placebo* OR doubl* NEAR/1 blind* OR singl* NEAR/1 blind* OR assign* OR allocat* OR volunteer*):de,ab,ti)<br>#5 #1 AND #2 AND (#3 OR #4)	133
**Web of Science**	#1 TI=(traditional Chinese medicine) OR TI=(complementary therapy) OR TI=(acupuncture) OR TI=(electro stimulation) OR TI=(electric stimulation) OR TI=(auricular) OR TI=(laser therapy) OR TI=(transcutaneous) OR TI=(acupoint application) OR TI=(acupoint stimulation) OR TI=(auricular point sticking) OR TI=(acupressure)<br>#2 TI=(smoking cessation) OR TI=(tobacco cessation) OR	44

	TI=(quit smoking) OR TI=(stop smoking) OR TI=(cease smoking) OR TI=(nicotine withdrawal) OR TI=(nicotine dependence) OR TI=(tobacco withdrawal syndrome)<br>#3 TI=(systematic review) OR TI=(review) OR TI=(meta-analysis) OR TI=(meta analysis)<br>#4 (((((((((((((((TS=(randomized)) OR AB=(randomized)) OR TS=(placebo)) OR AB=(placebo)) OR TS=(drug therapy)) OR TS=(randomly)) OR AB=(randomly)) OR TS=(trial)) OR AB=(trial)) OR TS=(groups)) OR AB=(groups)) OR TS=(crossover or cross-over)) OR TS=(pragmatic clinical trial)) OR TS=(randomized controlled trial)) OR TS=(controlled clinical trial)) NOT TS=(Animals)) NOT TS=(Humans)<br>#5 #1 AND #2 AND (#3 OR #4)	

Figures

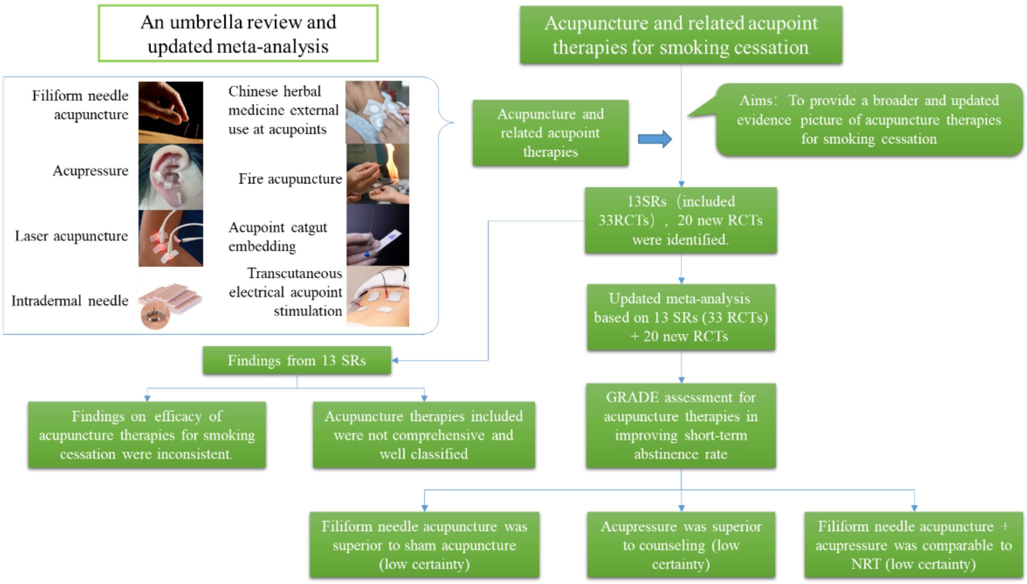

Figure 1. Graphical abstract. Notes: NRT, nicotine replacement therapy; RCT, randomized controlled trial; SRs, systematic reviews, GRADE, Grading of Recommendations Assessment, Development and Evaluation

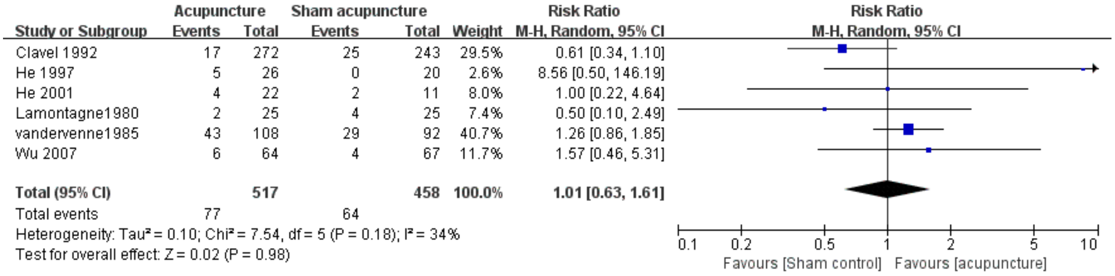

Figure 2. Filiform needle acupuncture *vs* sham acupuncture for long-term

smoking cessation

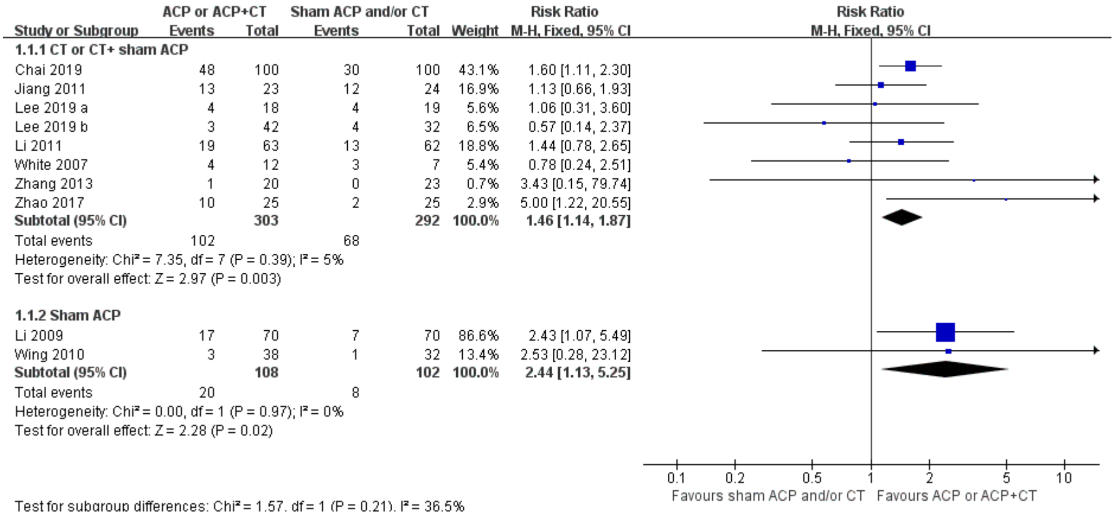

Figure 3. Acupressure for short-term smoking cessation
Notes: ACP, acupressure; CT, conventional therapy

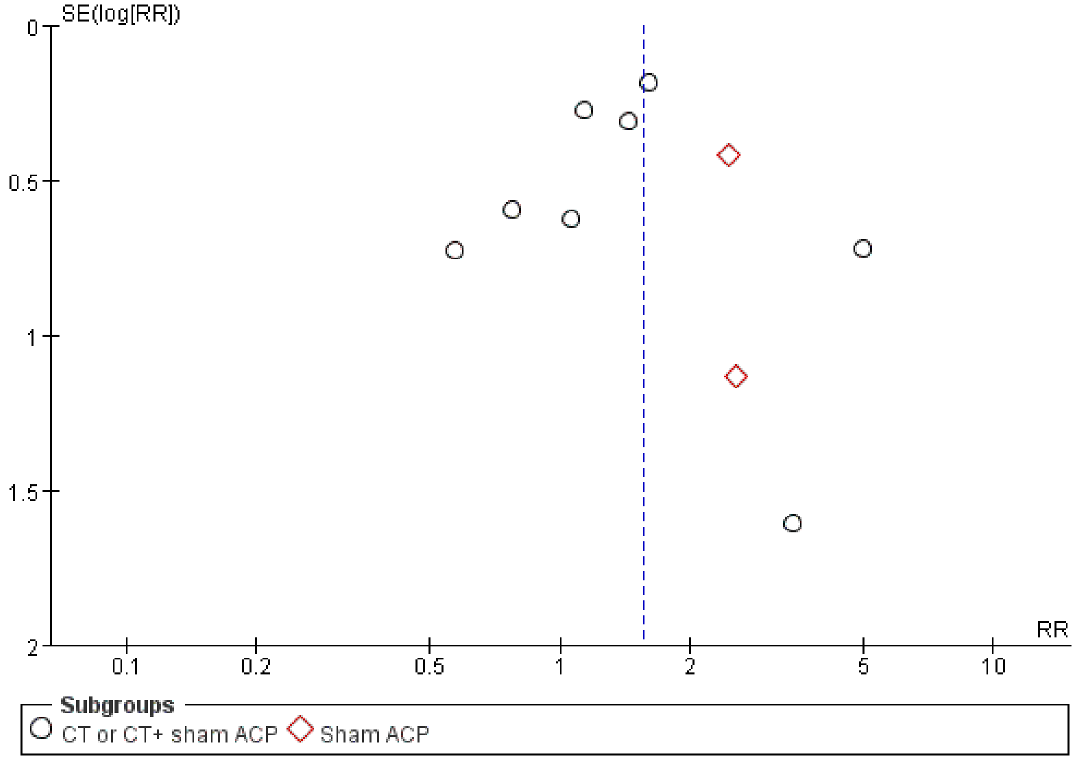

Figure 4. Funnel plot of acupressure for short-term smoking cessation

PRISMA 2009 Checklist

Section/topic	#	Checklist item	Reported on page #
**TITLE**			
Title	1	Identify the report as a systematic review, meta-analysis, or both.	1
**ABSTRACT**			
Structured summary	2	Provide a structured summary including, as applicable: background; objectives; data sources; study eligibility criteria, participants, and interventions; study appraisal and synthesis methods; results; limitations; conclusions and implications of key findings; systematic review registration number.	1-2
**INTRODUCTION**			
Rationale	3	Describe the rationale for the review in the context of what is already known.	2-3
Objectives	4	Provide an explicit statement of questions being addressed with reference to participants, interventions, comparisons, outcomes, and study design (PICOS).	3
**METHODS**			
Protocol and registration	5	Indicate if a review protocol exists, if and where it can be accessed (e.g., Web address), and, if available, provide registration information including registration number.	3
Eligibility criteria	6	Specify study characteristics (e.g., PICOS, length of follow-up) and report characteristics (e.g., years considered, language, publication status) used as criteria for eligibility, giving rationale.	3-4
Information sources	7	Describe all information sources (e.g., databases with dates of coverage, contact with study authors to identify additional studies) in the search and date last searched.	4
Search	8	Present full electronic search strategy for at least one database, including any limits used, such that it could be repeated.	**Supplementary file**

Study selection	9	State the process for selecting studies (i.e., screening, eligibility, included in systematic review, and, if applicable, included in the meta-analysis).	4
Data collection process	10	Describe method of data extraction from reports (e.g., piloted forms, independently, in duplicate) and any processes for obtaining and confirming data from investigators.	4
Data items	11	List and define all variables for which data were sought (e.g., PICOS, funding sources) and any assumptions and simplifications made.	4
Risk of bias in individual studies	12	Describe methods used for assessing risk of bias of individual studies (including specification of whether this was done at the study or outcome level), and how this information is to be used in any data synthesis.	4-5
Summary measures	13	State the principal summary measures (e.g., risk ratio, difference in means).	5
Synthesis of results	14	Describe the methods of handling data and combining results of studies, if done, including measures of consistency (e.g., I^2) for each meta-analysis.	5

Section/topic	#	Checklist item	Reported on page #
Risk of bias across studies	15	Specify any assessment of risk of bias that may affect the cumulative evidence (e.g., publication bias, selective reporting within studies).	5
Additional analyses	16	Describe methods of additional analyses (e.g., sensitivity or subgroup analyses, meta-regression), if done, indicating which were pre-specified.	5
**RESULTS**			
Study selection	17	Give numbers of studies screened, assessed for eligibility, and included in the review, with reasons for exclusions at each stage, ideally with a flow diagram.	5
Study characteristics	18	For each study, present characteristics for which data were extracted (e.g., study size, PICOS, follow-up period) and provide the citations.	6-7, **Table 1-2**
Risk of bias within studies	19	Present data on risk of bias of each study and, if available, any outcome level assessment (see item 12).	7
Results of individual studies	20	For all outcomes considered (benefits or harms), present, for each study: (a) simple summary data for each intervention group (b) effect estimates and confidence intervals, ideally with a forest plot.	7-10
Synthesis of results	21	Present results of each meta-analysis done, including confidence intervals and measures of consistency.	7-10
Risk of bias across studies	22	Present results of any assessment of risk of bias across studies (see Item 15).	7, **Figure 2**
Additional analysis	23	Give results of additional analyses, if done (e.g., sensitivity or subgroup analyses, meta-regression [see Item 16]).	9
**DISCUSSION**			
Summary of evidence	24	Summarize the main findings including the strength of evidence for each main outcome; consider their relevance to key groups (e.g., healthcare providers, users, and policy makers).	11-13
Limitations	25	Discuss limitations at study and outcome level (e.g., risk of bias), and at review-level (e.g., incomplete retrieval of identified research, reporting bias).	13
Conclusions	26	Provide a general interpretation of the results in the context of other evidence, and implications for future research.	13-14

FUNDING			
Funding	27	Describe sources of funding for the systematic review and other support (e.g., supply of data); role of funders for the systematic review.	14

From: Moher D, Liberati A, Tetzlaff J, Altman DG, The PRISMA Group (2009). Preferred Reporting Items for Systematic Reviews and Meta-Analyses: The PRISMA Statement. PLoS Med 6(6): e1000097. doi:10.1371/journal.pmed1000097

For more information, visit: **www.prisma-statement.org**.

©2024 Zhang Y.Y. et al.
